# Yeast‐insect associations: It takes guts

**DOI:** 10.1002/yea.3309

**Published:** 2018-02-22

**Authors:** Irene Stefanini

**Affiliations:** ^1^ Division of Biomedical Sciences University of Warwick Gibbet Hill Road Coventry CV4 7AL UK

**Keywords:** insects, interactions, symbiosis, yeasts

## Abstract

Insects interact with microorganisms in several situations, ranging from the accidental interaction to locate attractive food or the acquisition of essential nutrients missing in the main food source. Despite a wealth of studies recently focused on bacteria, the interactions between insects and yeasts have relevant implications for both of the parties involved. The insect intestine shows several structural and physiological differences among species, but it is generally a hostile environment for many microorganisms, selecting against the most sensitive and at the same time guaranteeing a less competitive environment to resistant ones. An intensive characterization of the interactions between yeasts and insects has highlighted their relevance not only for attraction to food but also for the insect's development and behaviour. Conversely, some yeasts have been shown to benefit from interactions with insects, in some cases by being carried among different environments. In addition, the insect intestine may provide a place to reside for prolonged periods and possibly mate or generate sexual forms able to mate once back in the external environments.

YEA‐May‐17‐0084.R3

## 1 INTRODUCTION

With almost 1,000,000 described species and approximately 6 million estimated total species, insects represent a large part of the biodiversity on Earth (Larsen, Miller, Rhodes, & Wiens, 2017). The insects we know most intimately are those which have a close relation, either positive or negative, with our lives. Insects may represent a pest (i.e. caterpillars causing crop damages), a vector of human pathogens (i.e. *Anopheles* spp., mosquitoes that trasmit malaria), a food resource, both as producer of food (i.e. honey) and as a food *per se* (i.e. termites and grasshoppers), as well as a pivotal resource for the maintenance of the natural biodiversity (as a consequence of plant pollination). Recently, pollinators such as honeybees (*Apis mellifera*) and bumblebees (*Bombus* spp.), which play an important role in human activities, have suffered a dramatic decline worldwide (Goulson, Nicholls, Botías, & Rotheray, 2015). Given its widespread occurrence and dramatic impact on the environment and human activities, pollinator decline soon became the object of many studies worldwide (Goulson et al., 2015). Aiming at the identification of the causes of the decline, investigations followed various paths, focusing on factors related to the environment, human intervention or microbial factors (Fairbrother, Purdy, Anderson, & Fell, 2014). One of the primary causes of the decline, probably the trigger, was the diffusion of *Varroa* spp. The mite, originated in Asia, spread across the world in 40 years, infecting and weakening adult bees by sucking their haemolymph and rapidly infecting the entire colony. Later on, new enemies, this time the microbes *Nosema ceranae (Higes, Meana, Bartolomé, Botías, & Martín‐Hernández,* 2013 *)* and *Ascosphaera apis (Aronstein & Murray,* 2010 *)* threatened pollinators. Both *Varroa destructor* and *Nosema* spp. (*N. ceranae* and *N. apis*) infestations have been shown to modify the composition of the insects’ gut microbiota (Hubert et al., 2017; Maes, Rodrigues, Oliver, Mott, & Anderson, 2016). In addition, *N. ceranae* infections of *A. mellifera* colonies can be controlled by treating the colony with fumagillin (Higes et al., 2008). This molecule, produced by the fungus *Aspergillus fumigatus* and used for control of *Nosema* disease in honey bees (Higes et al., 2011), avoids the bees’ colony collapse induced in untreated colonies by disrupting *N. ceranae*'s DNA replication (Hartwig & Przelecka, 1971; Higes et al., 2008; Huang, Solter, Yau, & Imai, 2013; Williams, Sampson, Shutler, & Rogers, 2008). These and other observations suggest the existence of a link between the microbial populations associated with healthy and affected insects and the outcome of the infestation. Hence, the impact of these new pathogens renewed the interest in uncovering the relationships between insects and microbes, aiming at the identification of the potential roles of microbes in controlling or favouring pathogen establishment (Alberoni, Gaggìa, Baffoni, & Di Gioia, 2016). The impact of bacterial communities present in the intestine of social insects has been widely explored (Kwong & Moran, 2016). Several yeasts are known to play a role in insects’ lives, aiding in food localization, contributing to food digestion or representing a valuable source of essential nutrients. Although the insect intestine may resemble a harsh environment, microorganisms are able to survive and possibly reproduce there, potentially setting up a long‐lasting association with their host. This review describes the most relevant known yeast–insect associations between ‘true yeasts’ (Saccharomycetes) (Kurtzman, Fell, & Boekhout, 2011) and insects, also reporting, where known, the establishment process and the benefits achieved by both insects and yeasts. Owing to their relevance, a few cases of insect associations with yeast‐like species (not belonging to the class Saccharomycetes) will be reported, i.e. *Symbiotaphrina* spp. (phylum Ascomycota, subdivision Pezizomycotina) and *Cryptococcus* spp. (phylum Basidiomycete).

**Outstanding questions about yeast–insect associations**

***Are there intestinal factors selecting for certain yeasts?***

The insect intestine is considered a hostile environment for many environmental microorganisms. However, neither the factors nor the extent to which the ingested yeasts are selected through the intestinal canal is known so far. Analysing the intestinal mycobiota by means of Next Generation Sequencing approaches on an extended set of insect species will be fundamental to identifying surivor yeasts and to disclosing the variation among fungal populations in different insect species/families.

***Does immunity play a role in the yeast–insect association?***

The host immune response is one of the factors potentially affecting the establishment of yeast–insect associations. Although many fundamentals on human immunology have been learned from the insect model (*Drosophila* spp.), variation in the insect's immune response to environmental microbes is still far from being fully known. In fact, the nature (positive or negative) of the effect of the interaction on host health is still under debate. To make the picture even more complicated, most social insects adopt a series of behaviours (i.e. grooming) which contribute to the control of potential pathogens. The use of *Drosophila* and *Galleria mellonella*, widely used in immunological studies, will be fundamental to gaining information on the variability of the immune response to a plethora of yeasts. However, because different insect species belonging to the same genus have shown different responses to the same fungi, further investigations on different insect species will be necessary.

***Can we completely uncover all yeast–insect associations?***

Considering the huge number of insect species, and the fact that our current knowledge is estimated to cover less than one‐fifth of the actual biodiversity, it is unlikely that we will ever be able to discover all of the possible associations between insects and yeasts. However, by further understanding already known associations, their mechanism of establishment and the full range of benefits or disadvantages for both players, we will be possibly able to formulate more general rules.

***What are we missing on the yeast benefits from the association?***

Until recently, vectoring and protection have been considered the only benefits gained by the yeast from their association with insects. However, the identification of new species found only in the insect intestine, and the assessment of the ability of yeasts to mate in this environment, recently expanded our knowledge. Still, we are probably missing other potential benefits for the yeast, such as the control of yeast competitors or the availability of an environment suitable for growth or survival in specific external conditions (i.e. the lack of exploitable substrates). Currently, it is hard to predict the extent of what we are missing, but further investigations on the extablished associations will surely help fill the gap.

## 2 THE INSECT INTESTINE: STRUCTURE AND ENVIRONMENTAL CHARACTERISTICS

**Figure 1:**
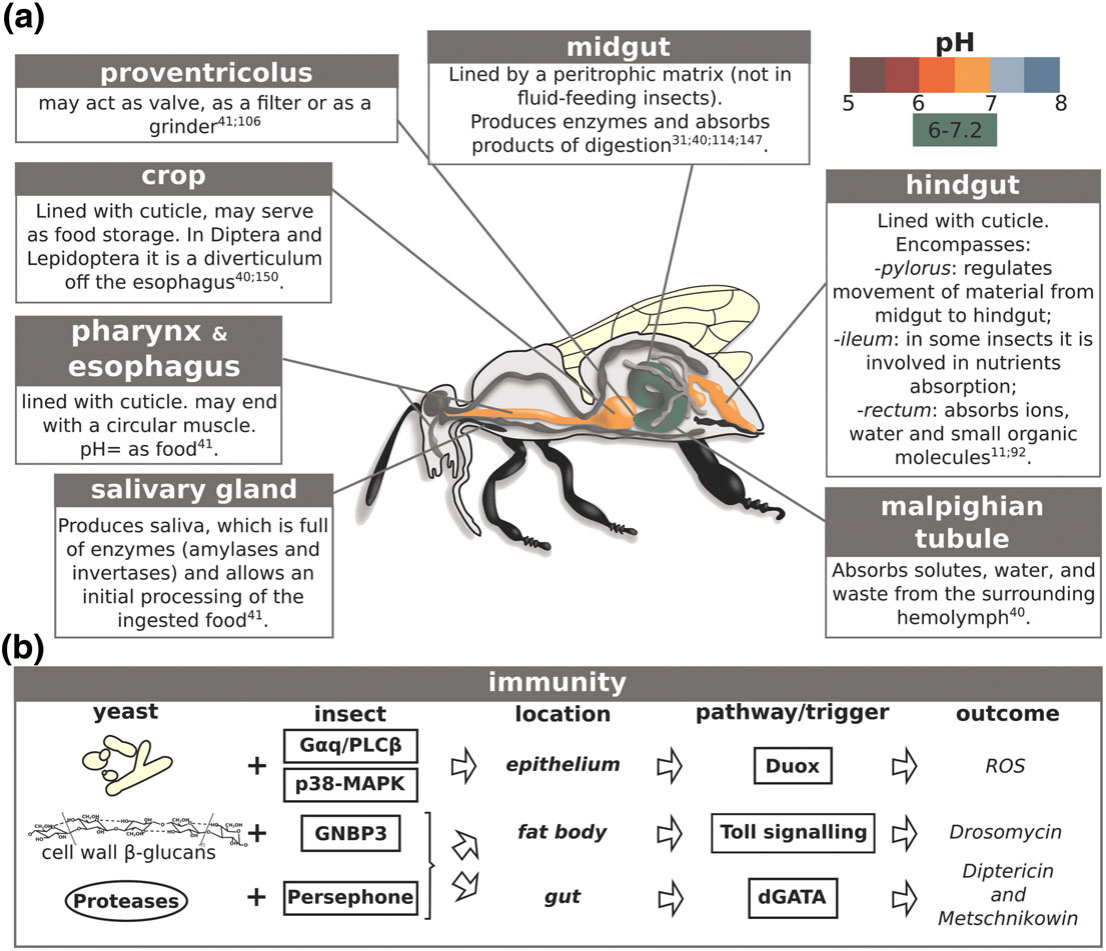
The internal anatomy of an insect. (a) Description of the parts composing the insect intestinal tract and their principal conformational and chemo‐physical characteristics. (b**)** The insect immunity involved in the recognition and clearance of external microbes, and of yeasts in particular. Superscript numbers refer to the reference as listed in the reference list [Colour figure can be viewed at http://wileyonlinelibrary.com]

The insect alimentary system normally consists of a continuous tube between the mouth and the anus. Its length varies according to the insect's feeding habits, usually shorter in carnivorous species and longer in phytophagous insects (Gillott, 2005a). In general, the alimentary canal consists of three regions: the foregut, the midgut and the hindgut. Each of these regions is dedicated to specific processes: the foregut is dedicated to food intake and storage, filtering and partial digestion; the midgut is the primary site of digestion and absorption; finally, in the hindgut, the absorption is completed and feces are formed (Fig. 1a) (Billingsley & Lehane, 1996).

### 2.1 The foregut

The salivary glands reside at the top of the entire canal and produce the saliva, a watery fluid rich in enzymes useful for the initial processing of the food (Gillott, 2005b). The enzymes present in the saliva widely vary among different insect species, according to the feeding habits, i.e. cellulose‐digesting enzymes in termites, fat‐digesting enzymes in carnivorous species (Gillott, 2005b). Once ingested, the food enters the foregut, where it undergoes initial processing. The foregut encompasses the pharynx, the oesophagus and the crop (Fig. 1a) and is lined with a cuticle that is shed at each moult (Chapman, 1998). The pharyngeal intima is surrounded by dilator muscles which are well developed in sucking insects, where they form the pharyngeal pump (Gillott, 2005b). The function of the proventriculus, located between the crop and the gut, varies in different insects. It may act as a valve, regulating the passage of food from the foregut to the midgut, as a filter, holding back the solid components of food, or as a grinder, breaking up the food into smaller pieces (Chapman, 1998). This particular structure has been shown to play a role in regulating the progression of microorganisms to the posterior intestine of some insects. As an example, in *A. mellifera*, the proventriculus filters particles smaller than 100 μm in diameter (Peng & Martson, 1986). Bigger particles and the fluid are excluded from the midgut and may be regurgitated with the nectar carried in the crop. By tracking the passage of several microorganisms through the proventriculus, Peng and Martison showed that microorganisms are included in the bolus and enter the midgut, where they can be digested, contribute to the digestion or temporarily reside (see further details below) (Peng & Martson, 1986). The filtering has several favourable outcomes: it excludes from the crop microorganisms which could contaminate and spoil the honey or infect larvae (i.e. *Bacillus larvae*), but it allows the digestion of nutrient yeasts (such as *Cyberlindnera jadinii*) (Peng & Martson, 1986). On the other hand, pathogenic fungi such as *Nosema apis* are allowed through and can thus infect the bee's midgut epithelial cells, finally resulting in the impairment of the insect's digestive functions (Peng & Martson, 1986). Conversely, in some insects (i.e. adult lacewings, *Chrysoperla* spp.), yeasts are more abundant in the crop (or foregut) than in the midgut or in the hindgut (Woolfolk & Inglis, 2004). In social insects, the crop acts as a food storage organ, a resource available to both the individual and other adults or larvae, which are fed by means of trophallaxis (Wainselboim & Farina, 2000). Generally, the pH of the foregut is the same as the ingested food, but in some insects it is <7 because of the presence of digestive microorganisms or of food regurgitated from the midgut (Gillott, 2005a).

### 2.2 The midgut

Unlike the foregut, the midgut is not lined with cuticle but it is lined by a peritrophic matrix (composed of proteins and chitin), which protects the gut epithelium against mechanical damage and external microorganisms (Terra, 2001) (Fig. 1a). The peritrophic matrix is generally absent in fluid‐feeding insects (i.e. Diptera and Lepidoptera), and its presence and/or composition may change throughout the life cycle of other insects (Gillott, 2005a). The midgut is usually a continuous structure, but in Hymenoptera three or four distinct regions are visible and dedicated to different functions: food storage, food movement, digestion and absorption (Gillott, 2005b). In some insects, mainly social insects feeding other adults or larvae by mean of trophallaxis, antiperistaltic movements move the digestive fluid from the midgut to the crop (Stoffolano & Haselton, 2013). Because the food is digested mostly in the midgut, the vast majority of the digestive enzymes are released there. Like salivary enzymes, the enzymes released in the midgut, besides liberating the nutrients from the ingested food, are also responsible for the death of sensitive ingested microorganisms (Terra, Ferreira, Jordao, & Dillon, 1996). Among these enzymes, lysozyme is responsible for the hydrolysis of the peptidoglycan present in the cell wall of many bacteria, while chitinases hydrolyse internal bonds in fungal cell wall chitin (Terra et al., 1996). The pH of the midgut varies among species, in general coinciding with the optimal value for the activity of the released enzymes (i.e. in wood‐feeding insects, the forepart of the midgut is 6.0–7.2, the optimum for amylases, while the posterior midgut has pH 9, the optimum for proteases) (Elpidina et al., 2001). An analysis of the malaria vector *Anopheles stephensi* clearly showed that this insect localizes cells of the yeast *Wickerhamomyces anomalus* in its midgut and gonads (Ricci et al., 2011). Notably, yeast cells were also found in the midgut of mosquitoes emerged in laboratory‐controlled conditions, suggesting a vertical transmission of *W. anomalus*, and persisted up to at least 10 days after the emergence, indicating the ability of this yeast to persist in the gut environment (Ricci et al., 2011).

### 2.3 The hindgut

The hindgut is lined with a cuticle like the foregut, but it is thinner because of the absorptive function of this portion of the intestine (Moussian, 2010) (Fig. 1a). The Malpighian tubules, the structures dedicated to the absorption of solutes, water and wastes from the haemolymph and to the production of uric acid, enter the gut in the hindgut (Beyenbach, Skaer, & Dow, 2010). The hindgut is composed of three regions: pylorus, ileum and rectum. The pylorus may be surrounded by a circular muscle regulating the movement of digested food from the midgut to the hindgut (Chapman, 1998). In general, the ileum has the function of transferring the food to the rectum, but in some insects water and ions absorption may occur here (Gillott, 2005c). The rectum is committed to the absorption of water, small organic molecules and ions, as well as the final production of feces. Owing to the presence of uric acid, the pH of the hindgut is typically 7. In this region, microorganisms are further selected: in some insects (such as wood‐eating insects), the ileum hosts a fermentation driven by microorganisms which use the uric acid released by the Malpighian tubules as a nitrogen source (Gillott, 2005a). Several different microorganisms, encompassing flagellated fermentative microorganisms, but also yeasts, inhabit the hindugt in different insects (Buchner, 1965; ega & Dowd, 2005). Peng and colleagues reported the digestion of *Candida utilis* cells (the anamorph of *Cyberlindnera jadinii*) in the alimentary canal of adult honeybee workers (*A. mellifera*) (Peng, Nasr, Marston, & Fang, 1984). By using histochemical approaches and observing the yeast morphology in several portions of the intestinal tract, Peng et al. showed that the digestion of yeast cells was accomplished by depolymerization of the cell wall (Peng et al., 1984). During the first hour after the ingestion of the yeast suspension, the morphology of yeast cells was not changed, and intact ellipsoidal yeast cells were observed in the midgut. Between 1 and 2 h after the ingestion, yeast cells located in the posterior part of the midgut showed a dramatically changed morphology (size increase, cylindrical shape, separation of the cell wall from the cytoplasm). After 3 h, many yeasts showed absent or partially broken cell walls. Finally, 15 h after the ingestion, the lack of staining of cytoplasmic proteins, glycogen and lipids in the honeybee worker rectum suggested that these components had been mostly digested and absorbed before entering the rectum. Only rare intact yeast cells were observed, clumped together and embedded in yeast debris.

### 2.4 The mycetome

In Dictyoptera, Hemiptera, Phthiraptera and Coleoptera, a special structure has been found to contain symbiont microbes: the mycetome (Douglas, 1989). This peculiar structure is composed of special cells, called mycetocytes, bigger than other insect cells and showing a cytoplasm cluttered by symbiotic microorganisms (Douglas, 1989). The mycetocyte symbionts are maternally inherited in most insects: a sole case of paternal inheritance was reported, in bostrychid beetles (Mansour, 1934). The maternal transmission of symbionts may occur through: (a) external smearing of the egg shell (i.e. yeast symbionts derived from the midgut caeca of anobiid beetles (Buchner, 1965)); (b) transovarial transmission (the symbionts are transferred from the mycetocytes to the ovary and then incorporated into the oocytes) (Douglas, 1989); or (c) the milk gland, a process observed in viviparous insects, i.e. Glossinidae (Aksoy, Chen, & Hypsa, 1997) and Hippoboscidae (Ma & Denlinger, 1974) (both Diptera). Mycetocytes may be free in the haemocoel, be associated with the intestinal tract or reside in the fat body, depending on the insect group. In most cases, the mycetome symbionts are bacteria, but a few cases of yeast symbionts have been documented (Noda, 1974; Spencer & Spencer, 1997). For example, *Coccidiascus legeri* is thought to be an intracellular symbiont of *Drosophila funebris* and *D. melanogaster (Spencer & Spencer,* 1997 *)*. Similarily, *Symbiotaphrina kochii* and *Symbiotaphrina buchneri* were found to be intracellular symbionts of *Stegobium paniceum* and *Lasioderma serricorne* (anobiid beetles) (Noda & Kodama, 1996). Whereas *Symbiotaphrina* (Pezizomycotina) is not a so‐called true yeast (where a ‘true yeast’ belongs to the subphylum Saccharomycotina), *C. legeri* has been regarded as a Saccharomycetales on the basis of its morphology (Kurtzman et al., 2011). Hence, the intracellular symbiosis seems to be limited to a few particular yeasts. However, it has to be considered that genetic analyses of some of the symbiontic yeasts (e.g. *C.s legeri*) were not possible owing to inability to culture them, and the current assignment of such yeasts, based only on their morphology, must be considered provisional (Kurtzman et al., 2011).

### 2.5 Immunity

For insects, as for other animals, the gut represents the route of entry for beneficial or detrimental (pathogenic) microorganisms. The intestine is the first defence against these microorganisms: it acts as a physical barrier, provides a hostile environment (mostly because of the pH and of the presence of lytic enzymes), and sets up an initial immune response (Lemaitre & Miguel‐Aliaga, 2013). *D. melanogaster* has proven to be a powerful model for the study of innate immunity (Hoffmann, 2003). The immune defence in *D. melanogaster* is based on two components: the humoural immunity (systemic), mediated by antimicrobial agents (AMP, antimicrobial peptides), and the cellular immunity, mediated by specialized cells present in the body cavity (Lu & St Leger, 2016). The cellular response relies on at least three differentiated blood cell types: plasmatocytes, lamellocytes and crystal cells. Plasmatocytes, representing the large part of all haemocytes, are responsible for the phagocytosis of microorganisms and are also involved in the mediation of the humoural response: their elimination abolishes AMP expression (Lu & St Leger, 2016). Several receptors are involved in the recognition of pathogen microbes by plasmatocytes. The most studied receptors are Eater (Kocks et al., 2005) and Dscam (Down syndrome cell adhesion molecule) (Graveley et al., 2004). Dscam has more than 12,000 potential splice variants, thus potentially providing precise recognition of specific pathogens (Graveley et al., 2004). However, so far the real potential of this receptor to recognize and bind fungal components has not been shown (Lu & St Leger, 2016). Similarly, to date Eater has not been shown to play a role in resisting fungi (Lu & St Leger, 2016). Concerning the humoural response, the NADPH oxidase dual oxidase 1 (Duox) is one of the immunological effectors against ingested microbes. It is indirectly activated by the presence of microbes through the G*α*q/phospholipase‐C*β* (PLC*β*) pathway or by the p38‐MAPK pathway downstream of the peptidoglycan receptor PGRC‐LC and Imd (Kim & Lee, 2014) (Fig. 1b). In the absence of Duox, G*α*q or PLC*β*, even dietary *Saccharomyces cerevisiae* cells can kill *Drosophila* flies (Ha et al., 2009). In turn, Duox is responsible for the production of reactive oxygen species, also contributing to microbial eradication (Welchman, Aksoy, Jiggins, & Lemaitre, 2009) (Fig. 1b). The yeast cell wall *β*‐glucans are recognized in the gut through binding by the GNBP3 receptorg (Gottar et al., 2006). Alternatively, yeast proteases induce Persephone maturation, another effector of the immune response (Gottar et al., 2006). Both of these signals trigger the Toll signalling pathway, which induces the expression of the antimicrobial agent Drosomycin in the insect fat body(Buchon, Silverman, & Cherry, 2014). (Fig. 1b). Recent studies suggest the existence of a tissue‐specific immune response in *Drosophila* gut, with dGATAe (a member of the GATA transcription factors) regulating antimicrobial gene expression (Diptericin and Metschnikowin) in the midgut independently from the Toll pathway(Senger, Harris, & Levine, 2006) (Fig. 1b). It is worth mentioning that most experiments investigating the response of *D. melanogaster* to yeasts used laboratory strains of *S. cerevisiae*, a species rarely found with natural *Drosophila* spp. populations (see above). Hence, these experiments may not be fully representative of the immune response mounted by insects against yeasts in nature. Aiming at the evaluation of possible bias owing to the use of laboratory *S. cerevisiae* strains, a recent study compared the response of adult *D. melanogaster* with a *S. cerevisiae* strain used for wine fermentation and *Hanseniaspora occidentalis*, *H. uvarum*, *Saccharomyces paradoxus*, *Brettanomyces naardenensis* and *Debaryomyces hansenii* isolated from wild *Drosophila* spp. insects (Hoang, Kopp, & Chandler, 2015). Hoang and colleagues showed that the differences among yeast species persistence are associated with the strain's resistance to reactive oxygen species (produced in the insect through the Duox response pathway(Welchman et al., 2009)), rather than to the origin of the strain (Hoang et al., 2015). The development of a *Drosophila* model to study intestinal infections by *Candida* spp. showed that the median time of flies’ survival upon injection of clinical *C. albicans* isolates was comparable with the survival of mice infected with the same yeast (Glittenberg, Silas, MacCallum, Gow, & Ligoxygakis, 2011). The use of this model revealed that *Candida albicans* triggered the expression of antimicrobial peptides in the fat body of the insect and induced an extensive JNK‐mediated death of insect's gut cells (Glittenberg et al., 2011). *Galleria mellonella* (Lepidoptera, known as greater wax moth or honeycomb moth) has been proposed as an additional model for the study of host–fungal interactions (Arvanitis et al., 2013). There are some advantages in using *G. mellonella* instead of *Drosophila* spp. to study the insects’ immune response to yeasts. First, *Galleria* is in general easier to handle (with no requirement for specialized equipment and experience). In addition, while wild‐type *G. mellonella* insects are sensitive to fungi (Lionakis, 2011) wild‐type *Drosophila* spp. insects are resistant to fungi, hence flies with perturbations in the Toll pathway need to be used (Alarco et al., 2004). *G. mellonella* was useful to discover several new features of the insect's immune response to yeasts. Among these, it has been shown that a pre‐exposure of *G. mellonella* larvae to *C. albicans* and *S. cerevisiae* cells, glucans from *S. cerevisiae* or laminarin (a polymer of *β*‐1,3 glucan from the alga *Laminaria digitata*) protects against a subsequent infection with a lethal inoculum of *C. albicans* (10^6^ cells) (Bergin, Murphy, Keenan, Clynes, & Kavanagh, 2006).

## 3 MICROBIAL COMMUNITIES AND INTERACTIONS

Despite several studies reporting the frequent identification of microorganisms (bacteria, fungi and protozoa) from insect intestines, their importance in food digestion and host health has been demonstrated for only a few insect species.

### 3.1 Drosophilids

**Figure 2:**
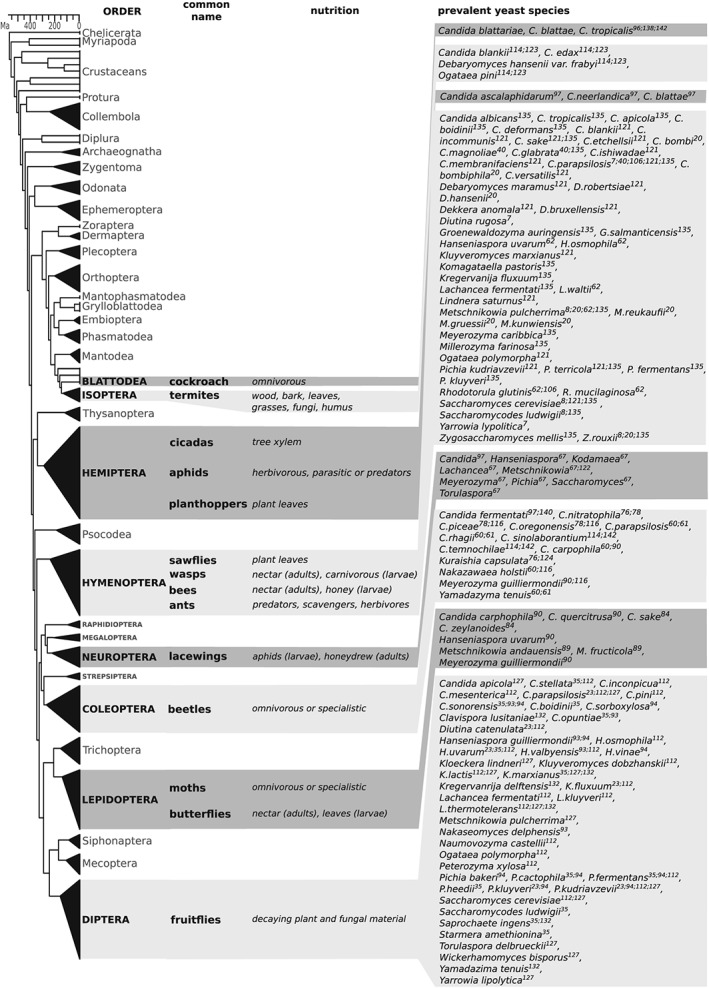
Known yeast–insect associations. Yeast species frequently found in the corresponding insect intestine. The insect phylogenetic tree has been adapted from Misof et al (Misof et al., 2014). Superscript numbers refer to the reference as listed in bibliography. Ma, Million years ago.

The *Drosophila* genus is probably the most studied insect from the behavioural, developmental and immunological viewpoints. Several studies, mainly focusing on bacteria, investigated the *Drosophila*–microbiome interactions (Broderick & Lemaitre, 2012). As a consequence, the relevance of yeasts in the development of *Drosophila* spp. is well known. In fact, the most commonly used laboratory *Drosophila* medium is based on yeast extract (Mitsuhashi, 1982). Noteworthy, despite *S. cerevisiae* being the yeast species mostly used in laboratory medium for *Drosophila* rearing, it has been rarely isolated from wild *Drosophila* intestines (Phaff, Miller, Recca, Shifrine, & Mrak, 1956). The yeast component of the *Drosophila* microbiota has been shown to encompass the yeast genera *Candida(Broderick & Lemaitre,* 2012 *)* [*C. apicola* (*Starmerella* clade)(Shihata & Mrak, 1952) *C. stellata* (*Starmerella* clade)(Fogleman, Starmer, & Heed, 1982; Phaff et al., 1956)], *C. inconspicua* (*Pichia* clade) (Phaff et al., 1956), *C. mesenterica* (*Kodamaea* clade) (Phaff et al., 1956), *C. parapsilosis* (*Lodderomyces‐Spathaspora* clade) (De Camargo & Phaff, 1957, Phaff et al., 1956; Shihata & Mrak, 1952); *C. pini* (Phaff et al., 1956), *C. sonorensis* (Fogleman et al., 1982; Morais, Hagler, Rosa, Mendonca‐Hagler, & Klaczko, 1992; Morais, Rosa, Hagler, & Mendonca‐Hagler, 1994), *C. boidinii* (*Ogataea* clade) (Fogleman et al., 1982), *C. sorboxylosa* (Morais et al., 1994), *Clavispora* (*C. lusitaniae* (Starmer, Heed, Miranda, Miller, & Phaff, 1976), *C. opuntiae(Fogleman et al.,* 1982 *; Morais et al.,* 1992 *)*), *Diutina* (*D. catenulata(De Camargo & Phaff,* 1957 *; Phaff et al.,* 1956 *)*), *Hanseniaspora(Broderick & Lemaitre,* 2012 *)* (*H. guilliermondii* (Morais et al., 1992, Morais et al., 1994); *H. osmophila* (Phaff et al., 1956), *H. uvarum(De Camargo & Phaff,* 1957 *; Fogleman et al.,* 1982 *; Phaff et al.,* 1956 *)* and its anamorph *Kloeckera apiculata* (De Camargo & Phaff, 1957; Morais et al., 1994; Phaff et al., 1956), *H. valbyensis* (Morais et al., 1992; Phaff et al., 1956), *H. vinae(Morais et al.,* 1994 *)*), *Kloeckera* (*K. lindneri(Shihata & Mrak,* 1952 *)*), *Kluyveromyces(Broderick & Lemaitre,* 2012 *)* (*K. dobzhanskii* (Phaff et al., 1956), *K. lactis* (Phaff et al., 1956; Shihata & Mrak, 1952), *K. marxianus(Fogleman et al.,* 1982 *; Shihata & Mrak,* 1952 *; Starmer et al.,* 1976 *)*), *Kregervanrija* (*K. delftensis(Starmer et al.,* 1976 *)*, *K. fluxuum(De Camargo & Phaff,* 1957 *; Phaff et al.,* 1956 *)*), *Lachancea* (*L. fermentati* (Phaff et al., 1956), *L. kluyveri* (Phaff et al., 1956), *L. thermotolerans(Phaff et al.,* 1956 *; Shihata & Mrak,* 1952 *; Starmer et al.,* 1976 *)*), *Metschnikowia* (*M. pulcherrima(Shihata & Mrak,* 1952 *)*), *Nakaseomyces* (*N. delphensis(Morais et al.,* 1992 *)*), *Naumovozyma* (*N. castellii(Phaff et al.,* 1956 *)*), *Ogataea* (*O. polymorpha(Phaff et al.,* 1956 *)*), *Peterozyma* (*P. xylosa(Phaff et al.,* 1956 *)*), *Pichia(Broderick & Lemaitre,* 2012 *)* (*P. barkeri* (Morais et al., 1994), *P. cactophila* (Fogleman et al., 1982; Morais et al., 1994), *P. fermentans* (Fogleman et al., 1982; Morais et al., 1994; Phaff et al., 1956), *P. heedii* (Fogleman et al., 1982), *P. kluyveri* (De Camargo & Phaff, 1957; Morais et al., 1994), *P. kudriavzevii* (De Camargo & Phaff, 1957; Morais et al., 1994; Phaff et al., 1956; Shihata & Mrak, 1952), *P. membranifacienss(Starmer et al.,* 1976 *)*), *Saccharomyces(Broderick & Lemaitre,* 2012 *)* (*S. cerevisiae(Phaff et al.,* 1956 *; Shihata & Mrak,* 1952 *)*), *Saccharomycodes* (*S. ludwigii(Fogleman et al.,* 1982 *)*), *Saprochaete* (*S. ingens(Fogleman et al.,* 1982 *; Starmer et al.,* 1976 *)*), *Starmera* (*S. amethionina(Fogleman et al.,* 1982 *)*), *Torulaspora* (*T. delbrueckii(Shihata & Mrak,* 1952 *)*), *Wickerhamomyces* (*W. bisporus(Shihata & Mrak,* 1952 *)*), *Yamadazima* (*Y. tenuis(Starmer et al.,* 1976 *)*) and *Yarrowia* (*Y. lipolytica(Shihata & Mrak,* 1952 *)*) (Fig. 2). The yeast species isolated from *Drosophila* intestines dramatically vary among different insect species or genetic backgrounds, thus leading to the hypothesis that the habitat partitioning (different *Drosophila* species share the same environment by feeding on different sources) can be influenced by yeast populations (Starmer & Fogleman, 1986). This hypothesis was reinforced by Lachance et al., who were able to predict the identity of the insect species on the basis of the phenotypes of yeasts isolated from their guts (Lachance, Gilbert, & Starmer, 1995)

### 3.2 Hymenoptera

Hymenoptera are another order of insects receiving particular attention in recent years, especially owing to its connection with human activities. Several studies investigated the relationship between honeybees (*Apis* spp., Fig. 2) and their microbiota, aiming at understanding the basis and eventually stemming the insects’ dramatic decline (Goulson et al., 2015). Such studies focused mainly on bacteria (Engel & Moran, 2013), but the relevance of yeasts in honeybees’ health has been known for a long time. In fact, it is common beekeeping practice to feed bees with baker's yeast in order to stimulate colony growth at the end of summer, and in 1919, Sackett reported the isolation of yeasts (*Saccharomyces* spp.) from adult honeybee intestines (Sackett, 1919). In 1987, Phaff and Starmer reported the isolation of hundreds of yeast strains from bee guts, belonging to over 20 different species (Phaff & Starmer, 1987). The large number and variability of isolates led the authors to the conclusion that the yeast presence could not be accidental. However, yeasts were thought to originate from food both because the intestinal yeast species showed strong seasonal variability and because nectar‐collecting bees bore different yeast species compared with pollen‐collecting species (Phaff & Starmer, 1987). Later on, Lachance et al. observed that yeasts found in the intestines of solitary bees (*Trigona* spp. and belonging to the Anthophoridae family) differ from these isolated from beetle intestines, thus suggesting the possibility of functional relationships (Starmer & Lachance, 2011). Interestingly, controlled experiments showed that honeybees treated with antibiotics bore a higher amount of yeasts, thus suggesting that bacteria usually compete with yeasts in the intestine (Gilliam & Prest, 1977). Similarly, stressed bees showed higher amounts of yeasts, but it is not clear whether this is a consequence or a cause of the stress (Gilliam, Wickerham, Morton, & Martin, 1974). Several different yeast species have been isolated from the intestine of *Apis* spp. (*A. cerana*, *A. mellifera*, *A. florea*, *A. indica*, *A. dorsata*), including *Candida blankii* (Sandhu & Waraich, 1985), *C. incommunis* (Sandhu & Waraich, 1985), *C. sake* (unaffiliated clade) (Sandhu & Waraich, 1985), *C. etchellsii* (Sandhu & Waraich, 1985), *C. magnoliae* (*Starmerella* clade) (Gilliam et al., 1974), *C. glabrata* (*Nakaseomyces* clade) (Gilliam et al., 1974; Stefanini et al., 2012), *C. ishiwadae* (*Nakazawaea* clade) (Sandhu & Waraich, 1985), *C. membranifaciens* (*Yamadazyma* clade) (Sandhu & Waraich, 1985), *C. parapsilosis* (*Lodderomyces*‐*Spathaspora* clade) (Gilliam et al., 1974;Sandhu & Waraich, 1985 ; Stefanini et al., 2012), *C. versatilis* (*Wickerhamiella* clade) (Sandhu & Waraich, 1985), *Dekkera anomala* (Sandhu & Waraich, 1985), *Dekkera bruxellensis* (Sandhu & Waraich, 1985), *Kluyveromyces marxianus* (Sandhu & Waraich, 1985), *Komagataella pastoris* (Stefanini et al., 2012), *Lindnera saturnus* (Sandhu & Waraich, 1985), *Metschnikowia pulcherrima* (Batra, Batra, & Bohart, 1973), *Ogataea polymorpha* (Sandhu & Waraich, 1985), *Debaryomyces maramus* (Sandhu & Waraich, 1985), *Debaryomyces robertsiae* (Sandhu & Waraich, 1985), *Pichia kudriavzevii* (Sandhu & Waraich, 1985). *Pichia terricola(Sandhu & Waraich,* 1985 *; Stefanini et al.,* 2012 *)* and *S. cerevisiae* (Batra et al., 1973; Sandhu & Waraich, 1985) Other bees have been found to bear yeasts in their intestines: the eusocial *Halictus* spp. bees (*Candida blankii*, *C. incommunis –* unaffiliated clade; *C. ishiwadae – Nakazawaea* clade)(Sandhu & Waraich, 1985) and the carpenter bees *Xylocopa* spp. (*Candida blankii –* unaffiliated clade; *C. versatilis – Wickerhamiella* clade; *C. ishiwadae – Nakazawaea* clade; *Crypotcoccus curvatus*, *Debaryomyces robertsiae*, *Pichia kudriavzevii*, *Pichia terricola*, *S. cerevisiae*) (Sandhu & Waraich, 1985). The association between yeasts and insects has also been studied in bumblebees (*Bombus*, Hymenoptera, Fig. 2), as relevant and endangered as honeybees. The yeast species which predominated in the microbiota of bumblebees were *Metschnikowia reukaufii*, *M. gruessii*, *M. pulcherrima*, *Metschnikowia kunwiensis*, *Candida bombi* (*Starmerella* clade), *C. bombiphila* (*Wickerhamiella* clade), *D. hansenii* and *Zygosaccharomyces rouxii (Brysch‐Herzberg,* 2004 *)*. Furthermore, the associations between yeasts and wasps (Hymenoptera) have also been recently assessed. The yeasts isolated from Vespidae intestines belonged to the genera *Candida* (*C. apicola(Stefanini et al.,* 2012 *)* – *Starmerella* clade; *C. boidinii – Ogataea* clade (Stefanini et al., 2012); *C. deformans* – *Yarrowia* clade (Stefanini et al., 2012); *C. sake(Stefanini et al.,* 2012 *)* – unaffiliated clade; *C. albicans(Stefanini et al.,* 2012 *)* and *C. tropicalis – Lodderomyces‐Spathaspora* clade(Stefanini et al., 2012; Suh, Nguyen, & Blackwell, 2008)), *Groenewaldozyma* (*G. auringiensis* and *G. salmanticensis*) (Stefanini et al., 2012), *Komagataella* (*K. pastoris(Stefanini et al.,* 2012 *)*), *Kregervanrija* (*K. fluxuum(Stefanini et al.,* 2012 *)*), *Metschnikowia* (*M. pulcherrima(Batra et al.,* 1973 *; Jimenez et al.,* 2017 *; Stefanini et al.,* 2012 *)*), *Meyerozyma* (*M. caribbica(Stefanini et al.,* 2012 *)*), *Millerozyma* (*M. farinosa(Stefanini et al.,* 2012 *)*), *Pichia* (*P. fermentans* and *P. kluyveri*)(Stefanini et al., 2012), *Saccharomyces* (*S. cerevisiae(Batra et al.,* 1973 *; Stefanini et al.,* 2012 *)*), *Saccharomycodes* (*S. ludwigi*i(Batra et al., 1973; Stefanini et al., 2012)) *Zygosaccharomyces* (*Z. mellis(Stefanini et al.,* 2012 *)* and *Z. rouxii(Batra et al.,* 1973 *; Stefanini et al.,* 2012 *)*), *Hanseniaspora* (*H. uvarum* and *H. osmophila*) (Jimenez et al., 2017), *Lachancea* (*L. fermentati* (Stefanini et al., 2012), *L. waltii(Jimenez et al.,* 2017 *)*) and *Rhodotorula* (*R. glutinis* and *R. mucilaginosa*) (Jimenez et al., 2017) Interestingly, only a small fraction of the species isolated in two studies on Vespidae collected in Italy(Stefanini et al., 2012) were also found in insects caught in Canada (Jimenez et al., 2017), suggesting either a geographic differentiation or a host specificity at the species level. Ants (Formicidae, Hymenoptera; Fig. 2) represent a particular case of renewed fungal–insect association. In particular, fungus‐farm ants (Attini) represent a great example of obligate mutualism with basidiomycetous fungi, which are cultivated by the ants as food (Mueller & Rabeling, 2008). Despite the association with mycelial basidiomycetous fungi being well established, evidence for a yeast–ant relationship is equivocal (Ganter, 2006). Yeast species isolated from ants are usually the same as those found in other surrounding sources (soil), such as *Yarrowia lipolytica (Ba & Phillips,* 1996 *). Aureobasidium pullulans* (Pagnocca, Rodrigues, Nagamoto, & Bacci, 2008), *Candida parapsilosis* (Ba & Phillips, 1996; Pagnocca et al., 2008), *Candida guilliermondii* (the anamorph of *Meyerozyma guilliermondii*), *D. hansenii*, *Diutina rugosa* (Ba & Phillips, 1996), *Rhodotorula glutinis(Pagnocca et al.,* 2008 *)* and *Yarrowia lypolytica(Ba & Phillips,* 1996 *)* were also found in leaf‐cutting ants belonging to the *Atta laevigata* and *A. capiguara* species and in the red fire ant (*Solenopsis invicta*). Other yeasts commonly found in the soil (*Candida vini* – the anamorph of *Kregervanrija fluxuum*; *Rhodotorula minuta* and *Rhodotorula mucilaginosa*) were not found in the ants’ nests, probably excluded by ants’ behaviours and chemicals controlling the contaminants (i.e. weeding and grooming) (Ba & Phillips, 1996)

### 3.3 Isoptera

Termite–microbe interactions are often used as an example to illustrate biological symbiosis because they depend on mutualistic intestinal microbes for provision of nitrogen and assistance with metabolism (see below for further details) (Saxena, Bahadur, & Varma, 1993; Schäfer et al., 1996) Termites (Isoptera) are traditionally separated into ‘lower’ and ‘higher’ termites based on their symbionts (Kumari et al., 2013). Lower termites (Mastotermitidae, Kalotermitidae, Hodotermitidae, Termopsidae, Rhinotermitidae, and Serritermitidae families) harbour prokaryotes and flagellate protists in their guts, whereas higher termites (family Termitidae) lack the protist symbionts (Abe, Bignell, & Higashi, 2000). In lower termites, flagellate protists are fundamental for cellulose digestion(Ebert & Brune, 1997) and higher termites overcame the lack of protists thanks to modifications in their diets, the presence of other intestinal microorganisms in their guts or higher gut compartmentalization and alkalinity (Brune, 2014). Other higher termites culture in their nests a basidiomycete fungus, genus *Termitomyces*, which, by feeding on termite workers’ feces, supports the digestion of pre‐processed wood (Mueller & Gerardo, 2002). As for ants, the association between some termites and fungi is well known, but only a few reports on yeast–termite associations are available. Large amounts of yeast belonging to the genera *Candida* (*C. blankii*, *C. edax* – the anamorph of *Sugiyamaella smithiae*), *Cryptococcus* (a Basidiomycete), *Debaryomyces* (*D. hansenii var. frabyi* – the teleomorph of *Candida farinata* v*ar. flareri*) and *Ogataea* (*O. pini*) were found in the gut of lower termites (between 10^7^ and 5 × 10^8^ cells per mL) (Prillinger & König, 2006; Schäfer et al., 1996). Of note, other yeasts isolated from lower (*Neotermes castaneus* and *Neotermes jouteli* – Kalotermitidae family; *Zootermopsis angusticollis* and *Zootermopsis nevadensis* – Termopsidae family; *Mastotermes darwiniensis* – Mastotermitidae family; and *Reticulitermes santonensis* – Rhinotermitidae family) and higher (*Nasutitermes nigriceps*, Termitidae family) termites and belonging to the *Scheffersomyces* clade (*Scheffersomyces stipitis*, *S. segobiensis*, *Candida shehatae*, *C. ergatensis*, and *Enteroramus dimorphus*) were shown to produce enzymes able to degrade hemicellulose, thus contributing to wood digestion (Schäfer et al., 1996; Wenzel, Schönig, Berchtold, Kämpfer, & König, 2002)

### 3.4 Lepidoptera

A very small part of the studies on yeast–insect associations focused on butterflies and moths (Lepidoptera; Fig. 2). However, the interest in these insects is justified by the fact that some of them are well‐known pests for economically relevant crops (i.e. *Helicoverpa armigera* for cotton or *Ostrinia nubilalis* for millet). In a few studies exploring Lepidoptera intestines, yeasts belonging to the *Candida carpophila* (*Meyerozyma* clade) (Molnár, Wuczkowski, & Prillinger, 2008), *C. quercitrusa* (*Kurtzmaniella* clade) (Molnár et al., 2008), *C. sake* (unaffiliated clade), *C. zeylanoides* (*Kurtzmaniella* clade) (Mankowski & Morrell, 2004), *Hanseniaspora uvarum* (Molnár et al., 2008), *Metschnikowia andauensis* (Mitsuhashi, 1982), *Metschnikowia fructicola(Mitsuhashi,* 1982 *)* and *M. guilliermondii(Molnár et al.,* 2008 *)* species were found. Despite *Galleria mellonella* (Lepidottera) being nowadays widely used as a model in studies on immunity and fungal infections (Arvanitis et al., 2013), reports of isolation of yeasts naturally associated with this moth are missing.

### 3.5 Coleoptera

A wealth of studies investigated the interactions between yeasts and insects of the Coleoptera order (Fig. 2), among which the most studied are the so‐called bark beetles (Scolytinae, Coleoptera, Fig. 2), the ambrosia beetles (Platypodinae, Coleoptera, Fig. 2) and the flower beetles (Scarabidae, Coleoptera, Fig. 2). Both bark and ambrosia beetles are known pests, attacking live trees and threatening their survival. Some 95% of yeasts found in flower beetle intestines are Saccharomycotina (Lachance et al., 2001). The species found in flower beetles intestines (Fig. 2) included *Nakazawaea holstii* (Jones, Dowd, & Blackwell, 1999; Rivera et al., 2009), *Candida fermentati* (the anamorph of *Meyerozyma caribbica*) (Nguyen, Suh, & Blackwell, 2007; Suh & Blackwell, 2004), *Candida nitratophila* (Leufvén, Bergström, & Falsen, 1984; Lou, Lu, & Sun, 2014), *C. piceae(Lou et al.,* 2014 *; Rivera et al.,* 2009 *)* (*Ogataea* clade), *C. oregonensis* (*Clavispora* clade), *C. rhagii* (*Hyphopichia* clade) (Jones et al., 1999; Jurzitza, Kühlwein, & Kreger‐van Rij, 1960), *Yamadazyma tenuis*, *C. sinolaborantium*, *C. temnochilae* (*Yamadazyma* clade) (Ravella et al., 2011; Suh, Nguyen, & Blackwell, 2005), *C. parapsilosis* (*Lodderomyces‐Spathaspora* clade) and *C. carpophila* (*Meyerozyma* clade) (Jones et al., 1999; Molnár et al., 2008). In addition, yeasts of other genera have been also found associated with flower beetles: *Kuraishia capsulata* (Leufvén et al., 1984; Shifrine & Phaff, 1956), *Meyerozyma guillermondii(Molnár et al.,* 2008 *; Rivera et al.,* 2009 *)* and *Torulopsis buchneri* (*Symbiotaphrina buchneri*) (Bismanis, 1976; Grabner, 1954). Interestingly, it has been shown that, in the absence of the insect, the insect‐associated yeasts are not found in the flowers (Lachance et al., 2001). thus confirming the role of flower beetles in vectoring yeast cells. Extensive investigations carried out on nitidulid beetles (in particular those found in flowers) allowed the identification of strong associations with some *Candida* and *Metschnikowia* species (see below for further details).

## 4 ADVANTAGES OF YEAST–INSECT ASSOCIATIONS

Once the existence of an association is established, a step further must consist of identifying the nature of the relationship, with a focus on the effects on both participants. In the majority of cases, the association is neutral (none of the two participants benefit or suffer from the association), but sometimes the association can be mutualistic (positive for both participants), commensal (positive for one, neutral for the the other), amensal (negative for one, neutral for the other) or parasitic (negative for one, positive for the other)(Starmer & Lachance, 2011). In some of the associations mentioned in the previous paragraph, the nature of the relationship has been revealed, showing interesting outcomes for both or at least one of the participants. Our present knowledge is unbalanced towards the identification of the benefits gained by insects associated with yeasts, rather than the opposite. When considering the benefit of both fungi and insects from the association, it is worth mentioning the relationship between beetles in the family Anobiidae and *Symbiotaphrina* spp., the intracellular yeast‐like symbionts (not considered ‘true yeasts’ because they do not belong to the class Saccharomycetes) (Noda & Kodama, 1996). Species of Symbiotaphrina can grow in laboratory conditions, have been isolated as endophytes and are always present in anobiid intestines (Blackwell, 2017). They have been shown to provide nitrogen and vitamin to their hosts, to degrade the disaccharide cellobiose, and to produce lipase, *α*‐ and *β*‐ glucosidase, phosphatase and trypsin, which may help the host in digesting the food and detoxifying a variety of compounds (ega & Dowd, 2005). Symbiotaphrina species have been assigned to several different genera, until the discovery of a new species helped in placing Symbiotaphrina and the new species, *Xylona heveae*, in a clade within Xylonomycetes. *Xylona heveae* was found as an endophyte in the sapwood of Peruvian rubber trees, but it lacks the ability to degrade cellulose and lignin, essential traits for entering the plant. Considering the great genomic similarity of *X. heveae* to animal‐associated taxa such as *Symbiotaphrina kochii*, Gazis and colleagues suggested that *X. heveae* could be insect‐transmitted, providing an explanation for entry into the plant in the absence of suitable enzymes (Gazis et al., 2016). The following paragraphs will highlight some of the best known and intriguing effects of the association on either the insect or the yeast.

### 4.1 Insect benefits

By attracting insects to suitable food sources, yeasts play a relevant role even before the establishment of an association with the insect. The initial attraction of insects to food is usually dependent on olfactory stimuli (Gillott, 2005a), and yeasts are known to attract beetles (Coleoptera) through the production of fermentative volatiles (Ganter, 2006). In addition, different *S. cerevisiae* strains have been recently shown to attract *Drosophila melanogaster (Palanca, Gaskett, Günther, Newcomb, & Goddard,* 2013 *)*. By studying this phenomenon at the molecular level, Christiaens and co‐workers showed that the ability of *S. cerevisiae* strains to attract fruit flies is associated with the *ATF1* gene, responsible for the production of the attracting compounds (acetate esters) (Christiaens et al., 2014). A similar observation was documented by Schiabor and co‐workers, who observed that mitochondria play a pivotal role in *S. cerevisiae* strains’ ability to attract *D. melanogaster (Schiabor, Quan, & Eisen,* 2014 *)*. In particular, Schiabor et al. showed that natural *S. cerevisiae* strains with mitochondria produced higher levels of ethyl esters, and the production of these volatile compounds was affected by the nitrogen levels in the substrate, with syntetic media mimicking the nutritional composition of fruit being the best environment for esters production (Schiabor et al., 2014). Similarly, many nitidulid beetles (also called ‘sap beetles’), which feed on fermenting plant sap, are attracted by the volatiles produced by yeasts during fermentation (Nout & Bartelt, 1998). However, as shown by the multifaceted relationship between *Drosophila* and yeasts, the localization of food is not the only benefit for insects. The development of *Drosophila* larvae is strongly affected by the presence of yeast in the insect's diet (Becher et al., 2012; Tatum, 2014). Yeasts provide *Drosophila* with organic nitrogen, essential vitamins (i.e. thiamin and riboflavin) and lipids, also restoring the growth impairment suffered by sunlight‐exposed larvae (Bruins, Scharloo, & Thörig, 1991). In addition, *Drosophila* shows a preference for specific yeast species even at the larval stage (Morais et al., 1994). Nevertheless, multi‐cultures have been shown to improve the insect development compared with monocultures (Starmer & Aberdeen, 1990). Furthermore, the yeast also plays a relevant role in *Drosophila* reproduction. For this insect genus, the main indicator of mating success is the size of the male face and, among males with comparable wide faces, females were shown to prefer males with their faces contaminated with yeasts (Norry, Vilardi, Fanara, & Hasson, 1995). In addition, as a courtship ritual, males give the females a nuptial gift, and the presence of yeasts in the nuptial gift makes the female more fecund (Steele, 1986). Even during oviposition, yeasts play a relevant role, with most (not all) of the *Drosophila* species’ females preferring to lay the eggs in substrates presenting yeasts (Oakeshott, Vacek, & Anderson, 1989). The reason for the preference of fruit flies for substrates and mates contaminated with yeasts may be the production by yeasts of aromatic compounds, as already mentioned (Christiaens et al., 2014). Similarly to drosophilids, honeybees are also commonly considered to benefit from the presence of yeasts in their food. In fact, to support the colony development, beekeepers often feed recently harvested or after‐wintered beehives with sugar supplemented with baker's yeast. In addition, recent findings suggest that the presence of yeasts associated with bees also supports insects’ activities, such as the preparation of bee bread, a mix of pollen and honey prepared and stored as food reserve by worker bees. Not only were significant amounts of yeasts (*Starmerella meliponinorum*) found in the bread produced by the stingless meliponine bees, suggesting that they can grow on this substrate (Teixeira et al., 2003), but yeasts (*Saccharomyces* spp.) also made the bread more attractive to honeybees (Pain & Maugenet, 1966). All of these observations suggested a beneficial effect of yeasts on bees. However, because of the observation that the yeast populations are greater if associated with stressed or caged bees, the contribution of yeasts to bees health is still a matter of debate. The rare identification of yeasts in healthy queen bees(Gilliam & Prest, 1977) and the significant amounts of yeasts (from 10^4^ c.f.u. mL^−1^ to 10^6^ c.f.u. mL^−1^ in different specimens) found in healthy adults of a stingless bee (*Tetragonisca angustula*)(Teixeira et al., 2003) suggest that the responses to yeasts may differ in different *Apis* species. Even for ants, yeasts seem to provide fundamental nutrients. A controlled experiment carried out by Mankowski and Morrel(Mankowski & Morrell, 2004) showed that *Camponotus vicinus* adults fed a diet supplemented with *Debaryomyces polymorphus* strains isolated from worker ants were heavier than insects fed on the same diet lacking the yeast. In addition, Ba and Phillips reported that colonies from which yeast could be isolated were more vigorous than these not presenting yeasts (Ba & Phillips, 1996). Despite not identifying the substances specifically provided by yeasts, these findings suggest that yeasts represent a significant source of nutrients for ants. Recently, the identification of antagonistic interactions between fungal pathogens and garden yeasts of the leaf‐cutting ant *Atta texana* suggested that insects may exploit yeasts to control diseases (Rodrigues, Cable, Mueller, Bacci, & Pagnocca, 2009). *Bulleromyces albus* and *Cryptococcus magnus* inhibited the growth of *Syncephalastrum racemosum*, while *Candida membranifaciens* and other unidentified yeast‐like isolates inhibited the hyphal development of *Beauveria bassiana (Rodrigues et al.,* 2009 *)*. Yeasts also help the insect in digesting difficult substances, i.e. in termite intestines cellulose, hemicellulose and xylans present in the wood. In fact, despite termites being able to produce their own cellulase, endogenous hemicellulases have not been found (Prillinger & Varma, 2006). Thus, the termite microbiota should at least provide the enzymes necessary to digest hemicellulose. Fungi, bacteria and yeasts have been shown to contribute to the degradation of these wood components (Saxena et al., 1993; Schäfer et al., 1996). *In vitro* experiments showed that yeasts (*Scheffersomyces stipitis*, *Scheffersomyces segobiensis*, *Candida shehatae*, *C. ergatensis* and *Enteroramus dimorphus*, all members of the *Scheffersomyces* clade) isolated from termite intestines were able to digest hemicellulose and xylan (Schäfer et al., 1996; Suh, White, Nguyen, & Blackwell, 2004; Wenzel et al., 2002) Ambrosia beetles (Platypodinae, Coleoptera; Fig. 2) excavate tunnels in live or dead trees and grow fungal gardens there. These fungal gardens were also shown to encompass yeasts (*Candida kashinagacola*, *Ambrosiozyma* clade) able to digest the wood (Suh, Kim, Son, Seo, & Kim, 2013). Controlled experiments clearly showed that fungal garden yeasts represent a source of essential nutrients for the beetles, such as nitrogen, which is low in the wood (Martin, 1988). Another intriguing role of microorganisms in the life of insects is that played by both bacteria and yeasts in controlling bark beetles aggregation (Scolytinae, Coleoptera; Fig. 2). Pioneer bark beetles (either male or female, depending on the insect species) infest trees in a solitary fashion. The pioneers release sex pheromones, which attract other bark beetles. The pheromones are produced either by *de novo* synthesis(Blomquist et al., 2010) or through digestion. In the latter case, the bacterium *Bacillus cereus* has been shown to be responsible for the conversion into verbenol (the pheromone) of the monoterpene *α*‐pinene present in the tree resin (Brand, Bracke, Markovetz, Wood, & Browne, 1975). When the size of the insect population (adults and larvae) reaches the maximum that the tree can tolerate, the infesting beetles stop pheromone production and begin to produce a repellent, verbenone. Interestingly, it has been shown that, among the microorganisms present in the insect intestine, some *Candida* and *Kuraishia* species (*Candida nitratophila –* of the *Ogataea* clade; *Kuraishia capsulata*; and *Candida molischiana –* of the *Kuraishia* clade) are able to carry out the conversion of verbenol into verbenone, thus indicating a strict relation between the insect behaviour and the presence of yeasts (Leufvén et al., 1984). Yeasts also play a meaningful role in regulating interactions among insect species. A documented example of such a role involves honeybees (Hymenoptera, Apidae, *A. mellifera*), their parasite, the small hive beetle (Coleoptera, Nitulidae, *Aethina tumida*) and the yeast *Kodamaea ohmeri (Torto, Boucias, Arbogast, Tumlinson, & Teal,* 2007 *)*. When honeybee workers and guards sense danger, they release alarm pheromones, a complex blend of over 40 aliphatic and aromatic compounds encompassing isoamyl acetate, 2‐heptanone, isopentyl acetate and methyl benzoate (Hunt, 2007). Some components of the alarm pheromones act as attractants when at low concentrations, to recruit as many nestmates as possible to defend the nest, but at higher concentration the same compounds act as repellents, to stave off further potential threats (Hunt, 2007). Unfortunately, the honeybee parasite *Aethina tumida* is attracted by the alarm pheromone, thus thwarting the bee's effort to protect the colony. Interestingly, an *in vitro* assay showed that, when grown in pollen, *Kodamaea ohmeri*, the yeast vectored by the parasite beetle, produces high levels of isopentyl acetate, one of the major components of the honeybees’ alarm pheromones. Thus, when a beetle attacks a beehive, the threat causes the bees to produce the attracting alarm pheromone, but the newly vectored yeast adds to the signal, eventually facilitating the effective infestation of the nest by parasite beetles (Hunt, 2007)

### 4.2 Yeast benefits

The extent of the benefits accrued to yeasts from yeast–insect associations is still poorly understood. In general, it is thought that, thanks to insects, yeasts can be vectored among substrates and potentially protected from unfavourable environments. In fact, while bacteria and mycelial fungi disperse through the air, yeasts require vectors to move among different environments. The spreading in natural environments has been shown to occur thanks to the action of both large animals(Francesca, Canale, Settanni, & Moschetti, 2012) and insects (Christiaens et al., 2014; Goddard, Anfang, Tang, Gardner, & Jun, 2010; Palanca et al., 2013). In some cases, the dispersal to new environments may represent the only possibility of survival because yeasts tend to exploit and deplete the nutrients present in their natural living substrate (Suh & Blackwell, 2005b). In addition, the insect intestine may represent an environment suitable for yeast growth and survival, providing a regular source of nutrition (ega & Dowd, 2005). For example, the beetle intestine makes available a stock of xylose, otherwise rarely present in natural environments, and could thus be a nutrient‐rich habitat for yeast species that are able to ferment and assimilate this sugar (Jackson & Nicolson, 2002). Furthermore, the insect intestine could represent a favourable environment for some yeast species by limiting the number and variability of co‐occurring, potentially competing, microorganisms. The observation that the beetle gut usually hosts a single yeast species, as assessed both through microbe isolation(Suh & Blackwell, 2005b) and by cloning the LSU rRNA gene (Zhang, Suh, & Blackwell, 2003), possibly supports the hypothesis that the insect intestine regulates the composition of the resident yeast population. Recently, *S. paradoxus* was shown to be unable to survive in social wasp intestines, unless they formed hybrids with *S. cerevisiae (Stefanini et al.,* 2016 *)*. However, the social wasp intestine does not seem to select for specific traits at the intra‐species level (Dapporto et al., 2016). In contrast, one of the few identified intracellular symbionts, *C. legeri* (tentatively placed in the class of Saccharomycetales on the basis of its morphology(Phaff, 2011)), seems to live exclusively in association with the host insect. In fact, *C. legeri(Phaff,* 2011 *)* was observed in intestinal epithelial cells of *Drosophila funebris* and *D. melanogaster*, but could not be cultured in laboratory conditions in the absence of the insect cells (Spencer & Spencer, 1997). The environmental factors characterizing insect intestines and causing microbe survival or death have not yet been identified, and the reason why only a single or a few yeast species have been isolated is still unknown. It could be either that the prevalent yeast modifies the habitat to exclude other yeasts, or that the insect intestine selects for a particular yeast. In support of the latter hypothesis, a constant set of yeast species was isolated independently from more than one beetle at different life stages, indicating specificity at the host species level and the occurrence of vertical (or early) transmission (Suh & Blackwell, 2005b). Closely related yeast species belonging to the *Candida tanzawaensis* clade (recently reassigned to the *Suhomyces* genus) have been isolated from different beetle species of the same family, supporting the possibility of a horizontal transmission of yeasts, rather than the less likely existence of a common yeast ancestor shared by insects of the same family (Suh, McHugh, & Blackwell, 2004). Strong associations have been identified between yeasts of the large‐spored *Metschnikowia* clade and nitidulid beetles (Lachance et al., 2001; Lachance & Fedor, 2014). Most of the yeast species belonging to the large‐spored *Metschnikowia* clade have two relevant and peculiar characteristics, making them a noteworthy case of yeast–insect associations: they are endemic and show strict associations with insects, in particular with nitidulid beetles (Coleoptera: Nitidulidae) (de Oliveira Santos, Perri, Andrietta, Rosa, & Lachance, 2015). The first report of yeasts belonging to this clade was for *Metschnikowia hawaiiensis*, which is endemic to Hawaii (Lachance, Starmer, & Phaff, 1990). A subclade composed of species found mostly in association with *Conotelus* spp. beetles (Coleoptera: Nitidulidae) joins the *M. hawaiiensis* subclade. Interestingly, four major members of the yeast community found in Hawaiian *Conotelus* insects (*Metschnikowia ipomoeae*, *Metschnikowia kipukae*, *M. hawaiiensis* and *Metschnikowia lochheadii*) belong to the large‐spored *Metschnikowia* clade, but two of them (*M. ipomoeae* and *M. lochheadii*) were also found in Central America. These four species, despite being phenotypically nearly indistinguishable from one another, are evidently different at the genetic level, and Lachance and co‐workers provided evidence that *C. ipomoeae* and *M. lochheadii* were introduced to Hawaii through human activities (Lachance, Bowles, & Starmer, 2003). To date, the large‐spored *Metschnikowia* clade is continuously expanding thanks to the identification of new species associated with insects, mostly nitidulid beetles (de Oliveira Santos et al., 2015). The insect intestines may represent a peculiar environment for yeasts which cannot survive elsewhere. In fact, several new yeast species were discovered in the intestine of insects, particularly beetles (Masneuf, Hansen, Groth, Piskur, & Dubourdieu, 1998; Suh et al., 2005; Suh & Blackwell, 2004; Suh & Blackwell, 2005a; Suh, McHugh, & Blackwell, 2004). In addition, social wasp intestines have been recently shown to favour the intra‐ and inter‐species mating of *Saccharomyces* yeasts (Stefanini et al., 2016), further supporting the hypothesis that this environment could represent a source of yeast biodiversity. The presence of insects also affects the yeast biodiversity in the environment, by modifying both the density and the composition of yeasts populations. As previously mentioned, flower beetles have been shown to play a pivotal role in defining the composition of the flower's yeast communities, with flowers not visited by insects lacking several yeast species otherwise present (Lachance et al., 2001). In addition, *Drosophila* larvae were shown to reduce the differences among yeast populations on different fruits, also reducing the population density probably by discouraging the growth of moulds (Stamps, Yang, Morales, & Boundy‐Mills, 2012). In general, the association between insects and yeasts seems not to be fortuitous, even for the yeast counterpart. Three hypotheses of the origin of endosymbiotic yeast–insect associations have been proposed. The first suggested that symbionts were derived from insect commensals or pathogenic parasites (Steinhaus, 1949), while the second suggested that they were the descendants of phytopathogenic or saprophytic fungi (Dowd, 1991). A third hypothesis proposed that insect feeding habits led to the development of the association (ega & Dowd, 2005). Considering our limited knowledge so far on these associations, it is not surprising that mechanisms for adaptation remain elusive.

## 5 BIOTECHNOLOGICAL RELEVANCE

The knowledge of some yeast–insect associations has been useful in biotechnological applications. First, the well‐known ability of yeast to attract insects has been exploited to bait traps used to catch herbivorous insects (Davis & Landolt, 2013). Traps supplemented with live yeasts (*C. utilis*, the anamorph of *Cyberlindnera jadinii*) were more effective in catching pest fruit flies (Diptera) compared with traps containing the attracting chemicals only (Leblanc et al., 2010). Knight and co‐workers proposed exploiting one of the known yeast–insect associations as a biocontrol (Knight & Witzgall, 2013). Aiming at the control of the codling moth *Cydia pomonella*, a known apple tree pest, they combined a pathogen granulovirus with yeasts isolated from larvae. The treatment of apples with a combination of the virus with *M. pulcherrima* significantly increased the mortality of neonate insects compared with the treatment with the virus alone (Knight & Witzgall, 2013). More recently, the association between yeasts and *Drosophila* was exploited to reduce the insect fitness by means of RNA interference (Murphy, Tabuloc, Cervantes, & Chiu, 2016). In that study, Murphy and co‐workers showed that *S. cerevisiae* cells genetically modified to express a dsDNA were able to reduce locomotion and egg‐laying in adults and survival in larvae of *Drosophila (Murphy et al.,* 2016 *)*. The astonishing specificity of these effects, which affected the pest *Drosophila suzukii* but not *D. melanogaster*, highlights the great potential of this approach for the development of new biocontrol agents.

## 6 CONCLUSIONS

The association between yeasts and insects is only beginning to be understood. Our current knowledge recognizes the importance of these associations on the health and behaviour of the host and on yeast distribution in the environment. However, we are still far from completely understanding the rules governing these interactions and their effects on microbial and animal lives. So far, studies have focused primarily on the description of yeast communities associated with insects relevant to human activities (either for production, or as pests). Nevertheless, the discovery of the relations between yeasts and other insects will represent a fundamental step towards a better understanding of ecological and evolutionary interactions. The exploration will largely benefit from the use of metagenomics approaches to explore the composition of yeast communities. By describing the yeast populations associated with a wider range of insects, it will eventually be possible to assess species‐specific interactions. In addition, analyses of the physiology of yeasts found in these environments, from an insect‐benefit perspective, will further expand our knowledge. What is certainly missing so far is a better understanding of the benefits obtained by yeasts from the association with insects, thought to consist mainly of vectoring.

## CONFLICT OF INTEREST STATEMENT

The author declares that there is no conflict of interest
